# Severe Cushing’s syndrome due to small cell prostate carcinoma: a case and review of literature

**DOI:** 10.1530/EC-17-0081

**Published:** 2017-06-05

**Authors:** M S Elston, V B Crawford, M Swarbrick, M S Dray, M Head, J V Conaglen

**Affiliations:** 1 Department of EndocrinologyWaikato Hospital, Hamilton, New Zealand; 2 Waikato Clinical CampusUniversity of Auckland, Hamilton, New Zealand; 3 Department of RadiologyWaikato Hospital, Hamilton, New Zealand; 4 Department of PathologyWaikato Hospital, Hamilton, New Zealand; 5 Department of OncologyTauranga Hospital, Tauranga, New Zealand

**Keywords:** ectopic ACTH production, Cushing’s syndrome, small cell prostate cancer, neuroendocrine tumour

## Abstract

Cushing’s syndrome (CS) due to ectopic adrenocorticotrophic hormone (ACTH) is associated with a variety of tumours most of which arise in the thorax or abdomen. Prostate carcinoma is a rare but important cause of rapidly progressive CS. To report a case of severe CS due to ACTH production from prostate neuroendocrine carcinoma and summarise previous published cases. A 71-year-old male presented with profound hypokalaemia, oedema and new onset hypertension. The patient reported two weeks of weight gain, muscle weakness, labile mood and insomnia. CS due to ectopic ACTH production was confirmed with failure to suppress cortisol levels following low- and high-dose dexamethasone suppression tests in the presence of a markedly elevated ACTH and a normal pituitary MRI. Computed tomography demonstrated an enlarged prostate with features of malignancy, confirmed by MRI. Subsequent prostatic biopsy confirmed neuroendocrine carcinoma of small cell type and conventional adenocarcinoma of the prostate. Adrenal steroidogenesis blockade was commenced using ketoconazole and metyrapone. Complete biochemical control of CS and evidence of disease regression on imaging occurred after four cycles of chemotherapy with carboplatin and etoposide. By the sixth cycle, the patient demonstrated radiological progression followed by recurrence of CS and died nine months after initial presentation. Prostate neuroendocrine carcinoma is a rare cause of CS that can be rapidly fatal, and early aggressive treatment of the CS is important. In CS where the cause of EAS is unable to be identified, a pelvic source should be considered and imaging of the pelvis carefully reviewed.

## Introduction

Cushing syndrome (CS) secondary to ectopic adrenocorticotrophic hormone (ACTH)–producing tumours (EAS) accounts for approximately 10% of CS depending on the series (1). A variety of tumours have been reported to be associated with EAS. The most common are those of neuroendocrine origin, namely small-cell lung carcinoma (3.3–50%), bronchial carcinoid (4.8–38.9%), thymic carcinoid (4.7–10.6%), medullary thyroid carcinoma (MTC) (1.9–11.6%), gastroenteropancreatic neuroendocrine tumours (NET) (8.9–14%) and phaeochromocytomas (2.5–5.6%) (1, 2, 3).

Sources of ectopic ACTH production arising in the pelvis are rare although EAS has been described due to gonadal tumours (adenocarcinoma, androblastoma, Sertoli cell tumour, carcinoid tumour and teratoma) (4, 5, 6) and genitourinary sources including the prostate (7) as well as cloacogenic carcinoma of the anal canal (8). Prostate cancer causing CS due to ectopic ACTH or CRH production is rare with <30 published cases.

We describe a patient who presented with severe CS due to an ACTH-producing neuroendocrine carcinoma of the prostate and summarise previously reported cases.

## Case report

A 71-year-old Caucasian male was admitted to hospital with profound hypokalaemia, generalised oedema and new-onset hypertension. Further history established a two-week history of weight gain (~6 kg), generalised muscle weakness, labile mood and insomnia.

The patient’s medical history included a transurethral resection of the prostate 10 years earlier (histology not available), a recent colonoscopy and polypectomy of a hyperplastic caecal polyp.

Medications included hydrochlorothiazide. The patient had infrequently used a topical 0.1% triamcinolone dental cream over last 2–3 weeks for mouth ulcers but reported no oral, inhaled or parenteral corticosteroid use.

On examination, the patient was hypertensive (BP 154/74 mmHg), euphoric and grossly oedematous with bilateral pitting oedema to the mid-shins and mild facial oedema. The patient had oral candidiasis, thin skin, multiple petechiae on the chest, a small supraclavicular fat pad and proximal muscle weakness. There was no hyperpigmentation, moon facies, dorsocervical fat pad or abdominal striae.

Laboratory results showed a hypokalaemic metabolic alkalosis (Table 1). Hypercortisolaemia was confirmed with morning cortisol >1655 nmol/L, absence of diurnal cortisol rhythm and markedly increased 24-h urinary free cortisol excretion (24-h UFC).

**Table 1:** Pertinent laboratory investigations at initial assessment.

**Analyte**	**Result**	**Reference interval**
Serum potassium	2.3	3.5–5 mmol/L
Glucose	11.6	<7.8 mmol/L
HbA1c	33	<41 mmol/mol
Serum cortisol (1104 h)	>1655	200–700 nmol/L
Serum bicarbonate	36.3	22–26 mmol/L
Venous pH	7.498	7.35–7.45
PSA	1.35	<6.5 ng/mL
Initial endocrine evaluation		
Midnight cortisol	>1655	<50 nmol/L
1 mg overnight dexamethasone suppression test	>1655	<50 nmol/L
8 mg overnight dexamethasone suppression test	>1655	
24-h Urinary free cortisol	36,315	35–285 nmol/24 h
Plasma ACTH	72	2–11 pmol/L
Plasma CRH	0.7	<5 pmol/L
Chromogranin A	32	<20 U/L

The ACTH was elevated (72 pmol/L), confirming ACTH-dependent CS. MRI of the pituitary was normal, and there was failure to suppress cortisol secretion following both 1 mg and 8 mg overnight dexamethasone suppression tests, suggesting ectopic ACTH or CRH production rather than Cushing’s disease (CD).

Computed tomography (CT) of the chest, abdomen and pelvis was initially reported as normal. As no causative lesion was identified on imaging, the patient was transferred to a university hospital for further evaluation.

**Figure 1:**
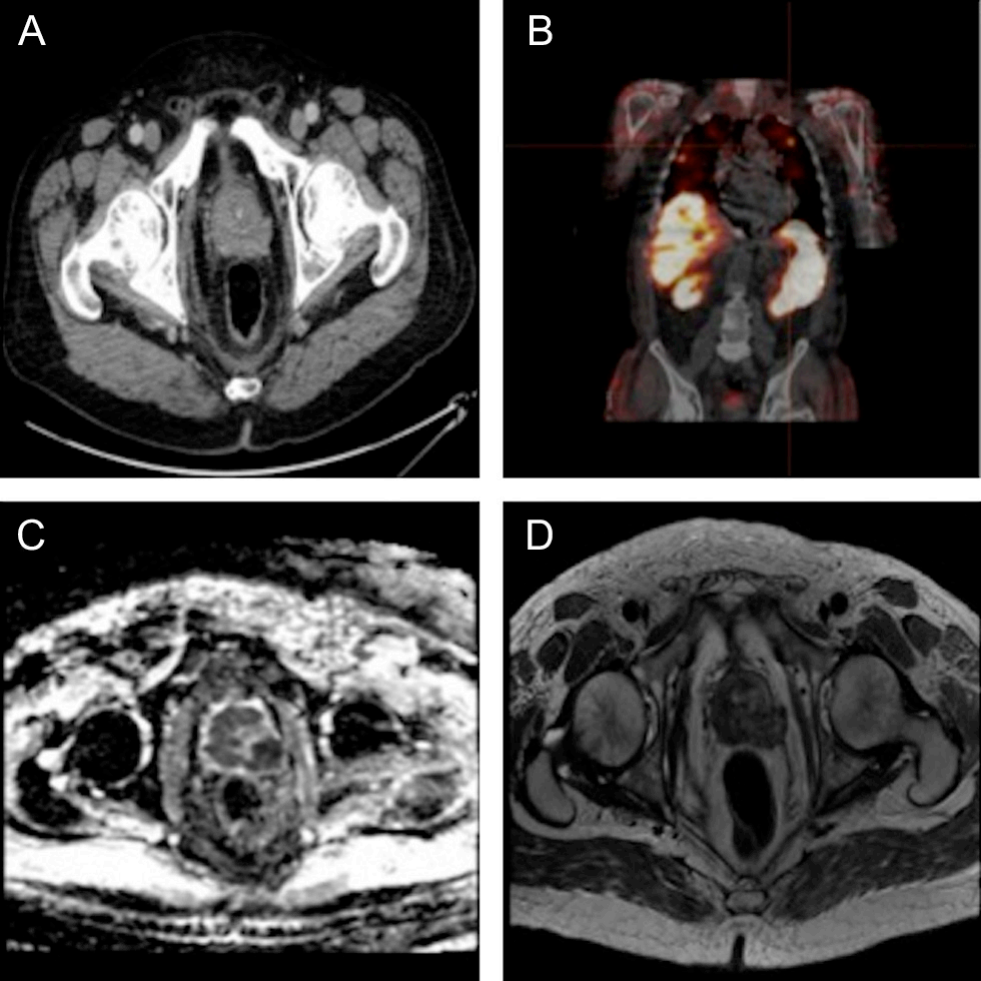
(A) CT imaging showing irregular prostate margins with possible extracapular spread. (B) Octreotide SPECT showing the presence of octreotide-avid pulmonary metastases. (C) Diffusion-weighted MRI imaging with ADC at 550 mm^2^/s. (D) T2-weighted MRI showing extensive signal abnormality of the prostate with extracapsular spread.

Careful review of the CT images detected an enlarged prostate with irregular margins and a soft tissue area in the pelvis felt to be possible abnormal lymph node enlargement (Fig. 1A). Additional history suggested mild symptoms of prostatism, and a hard irregular prostate gland was present on digital rectal examination. The patient’s prostate-specific antigen (PSA) was normal (Table 1).

An MRI pelvis revealed an enlarged prostate (52.6 cc volume), extracapsular spread of tumour, extensive T2 signal abnormality, with apparent diffusion coefficient (ADC) values of 550 mm^2^/s suggestive of high-grade prostatic malignancy (Fig. 1C and D). Abnormal bone marrow signal in pelvis and femur was consistent with bony metastases. A technetium-99^m^ 2,3-dicarboxypropane-1,1-diphosphonate isotope bone scan confirmed the presence of diffuse skeletal metastases and a technetium-99^m^-HYNIC-[Tyr3]-octreotide scan confirmed the presence of multiple pulmonary octreotide-avid lesions (Fig. 1B).

A CT-guided lymph node biopsy was inconclusive. Transrectal ultrasound-guided prostate biopsy was performed. Histological examination of the prostatic biopsy was consistent with a poorly differentiated prostate carcinoma with features of both high-grade (Gleason 5 + 5) conventional acinar adenocarcinoma (10–20% of the tumour) and a small-cell neuroendocrine carcinoma. Immunohistochemistry markers for neuroendocrine differentiation were positive (CD56, synaptophysin, neuron-specific enolase (NSE)), as was TTF-1. The Ki-67 (MIB-1) proliferation index was estimated as 35–45% (WHO grade 3) and weak positive staining for ACTH was present. Staining for CRH was not available. Plasma CRH levels were within the reference interval (Table 1). Sections of the tumour that did not demonstrate positive staining for neuroendocrine markers were positive for PSA.

**Figure 2:**
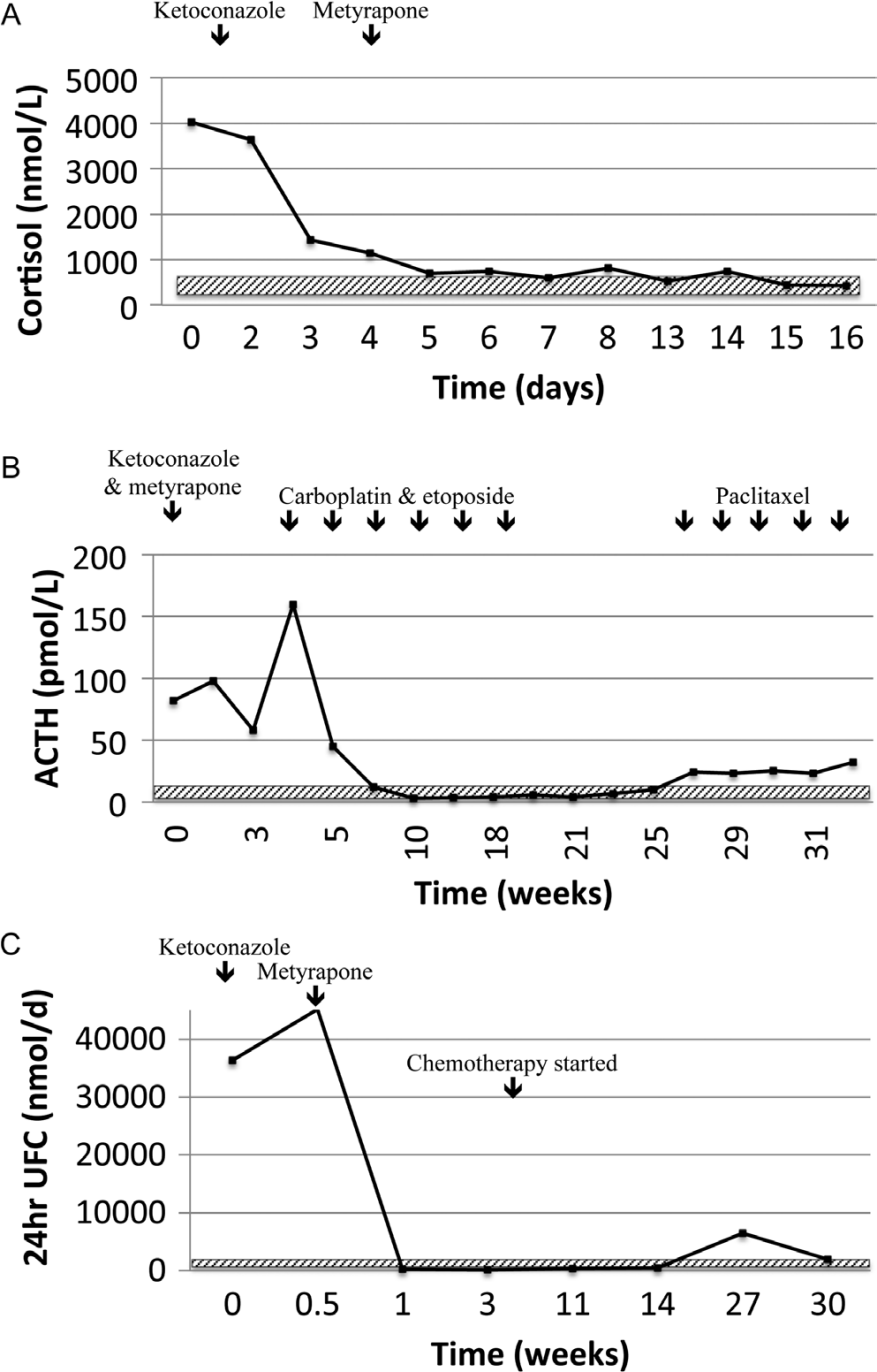
(A) Response of 08:00 h cortisol to adrenal blockade and chemotherapy. Cortisol reference interval 200–700 nmol/L (shown in hatched box). (B) Dramatic ACTH response to carboplatin and etoposide chemotherapy. ACTH reference interval 2–11 pmol/L (shown in hatched box). (C) 24-h urinary free cortisol response to adrenal blockade and chemotherapy. UFC = urinary free cortisol. 24-h UFC reference interval 35–285 nmol/24 h (shown in hatched box).

While awaiting histological confirmation of the diagnosis of prostate neuroendocrine carcinoma, urgent treatment with inhibitors of adrenal steroidogenesis was initiated (ketoconazole, 1.2 g daily in divided doses and metyrapone 6 g daily in divided doses), along with mineralocorticoid blockade (spironolactone) and potassium supplementation (Fig. 2A). The patient’s hypokalaemia was corrected and the 24-h urinary free cortisol normalised within one week (Fig. 2C).

The patient was commenced on chemotherapy targeting small-cell neuroendocrine carcinoma (carboplatin and etoposide) immediately after confirmation of histological diagnosis. Biochemical control of hypercortisolaemia was maintained and both ketoconazole and metyrapone were able to be discontinued. The patient completed four cycles of chemotherapy with radiographic evidence of disease regression and normalisation of ACTH, 08:00 h cortisol and 24-h UFC levels (Fig. 2A, B and C). By the end of six cycles, at five months following initial presentation, the patient developed evidence of further metastatic spread with increasing liver and bone metastases. Second-line treatment with paclitaxel was commenced, but recurrent hypercortisolism was evident by six months with a rise in morning cortisol to 722 mmol/L and ACTH to 19 pmol/L and 24-h urinary free cortisol to 2146 nmol/24 h (RR 35–285 nmol/day) requiring reinstitution of adrenal blockade. Due to continued disease progression, the patient was transferred to palliative care and died nine months after initial presentation. Ethical approval for case publication was provided by the institutional review board.

## Discussion

This case illustrated both diagnostic and treatment challenges. The patient presented with classical features of severe hypercortisolism typical of EAS with myopathy, hypertension, alkalosis, hypokalaemia and oedema. It was important to initiate urgent blockade of adrenal steroidogenesis plus blockade of the mineralocorticoid receptor while undertaking further investigations to determine the cause of the CS. Management of severe CS has recently been reviewed and while the optimal order of drug treatment for medical therapy is yet to be established, rapid blockade with ketoconazole and/or metyrapone, as used in this case, is recommended if parenteral therapy (etomidate) is not required (9). Other potential medical therapies to control hypercortisolism include mitotane, although the onset is slow, and mifepristone (not currently available in New Zealand) (10). Regular monitoring, both clinical and biochemical, is required to assess the efficacy of adrenal blockade and watch for the development of hypoadrenalism (10). In a multicentre study of 195 patients including 37 patients with EAS, where 164 patients received metyrapone monotherapy, biochemical monitoring showed improvement in all cases with approximately half of the patients developing normal cortisol levels and most of the adverse events mild and reversible (11). However, there were limited details on those with EAS in this paper making the relevance to this case unclear. Importantly, metyrapone may result in an increase in 11-deoxycortisol, which may cross-react with cortisol in cortisol immunoassays (12). Mineralocorticoid blockade was performed using spironolactone, as eplerenone was not available. Spironolactone may worsen androgen-sensitive prostate cancer as it may act as a selective androgen receptor modulator (13); however, in this case, the benefit of mineralocorticoid receptor blockade was considered to outweigh any potential harm.

From both the literature (14) and our personal experience, somatostatin analogues may be of benefit in EAS, but despite the tumour demonstrating uptake on somatostatin receptor scintigraphy, there was no biochemical response. A partial response to somatostatin analogue has previously been reported in CS secondary to prostate neuroendocrine carcinoma (15). While somatostatin receptor scintigraphy can be helpful to identify neuroendocrine tumours such as bronchial carcinoids causing CS, given the likely aggressive underlying tumour in this setting, FDG-PET/CT would have been more appropriate (16, 17). FDG-PET/CT was due to be performed in this case, but the diagnosis was confirmed prior to the scan being performed so the scan was cancelled as it was considered unlikely to change management.

Pelvic sources of CS are uncommon and the CT scan was initially reported as normal. This case demonstrates the importance of carefully reviewing imaging with an experienced radiologist. In addition, if a digital rectal examination was performed at initial assessment, it is probable that the diagnosis would have been made earlier; however, this would have been unlikely to alter the outcome. The prostate is not typically thought of as a cause for CS, as neuroendocrine tumours of the prostate are rare. Prostate neuroendocrine carcinoma of small cell type (SCPC) comprise approximately 0.5–2% of men with prostate carcinoma (18). Currently, it is thought that SCPC has a common origin with adenocarcinoma of the prostate (18). Approximately 40–50% of men with SCPC have a prior or concurrent history of prostatic adenocarcinoma (18).

Patients with SCPC typically present with lower urinary tract symptoms but may also have neurological symptoms due to brain metastases and/or pain from lytic bone lesions (19). Not only is SCPC associated with CS but also other paraneoplastic syndromes have been reported including hypercalcaemia, Eaton-Lambert and SIADH (18). PSA levels are typically normal, and SCPC is not responsive to androgen deprivation therapy (18). Given the rarity and lack of randomised trial data, treatment is typically based on that for small-cell lung cancer with platinum and etoposide-based chemotherapy (18). The molecular markers however differ with almost half of patients with SCPC demonstrating *ERG* (ETS transcription factor) rearrangements (similar to that of prostate adenocarcinoma) compared to an absence of this change in small-cell lung cancer. As such, assessment of *ERG* rearrangements may be helpful in determining a prostatic origin (20) but was not available in this case.

SCPC has an aggressive clinical course with median survival of 10–19 months despite platinum-based chemotherapy (18), which compares to approximately 60 months for patients with metastatic prostate adenocarcinoma (21). In patients who have CS associated with SCPC the prognosis is abysmal and, based on the currently published cases, the median survival is only two months (Table 2). Our patient survived for nine months from presentation, which is surprising given the markedly elevated levels of cortisol at presentation with peak 24-h urinary free cortisol >150-fold the upper limit of the reference range, and an extremely poor functional status when first transferred to our centre. The patient’s survival may be contributed to a prompt response to aggressive adrenal blockade and excellent initial response to chemotherapy. Of cases where the cause of death is reported, most patients die from sepsis, likely secondary to uncontrolled CS (Table 2). As such, control of the hypercortisolism is urgent and should not be delayed by waiting to identify the source of the CS. Patients who received both adrenal blockade and chemotherapy had a longer median survival compared to cases that only received adrenal blockade (including bilateral adrenalectomy) or no specific treatment (nine months vs two months and less than one month, respectively) (Table 2). In the current case, given the severity of the hypercortisolaemia, bilateral adrenalectomy was discussed, but was initially declined by the patient and then deferred due to a rapid response to chemotherapy. Bilateral adrenalectomy has been shown to be a potentially lifesaving procedure in patients critically unwell with CS (10, 22), although in the three cases of SCPC treated with bilateral adrenalectomy, death occurred at four weeks, three months and three weeks postoperatively due to metastatic disease, sepsis and liver failure from extensive metastatic disease, respectively (23, 24, 25).

**Table 2:** Summary of previous published cases of small-cell (neuroendocrine) prostate cancer.

Author	Year	**Patient age**	Stage	**Prior hx adenoCa**	**MR blockade**	CS treatment	Chemotherapy	**Survival from CS dx**	Cause of death
Webster (7)	1959	68	Metastatic	2 months prior	No	No	No	<1 week	Sepsis
Wise (23)	1965	58	Metastatic	38 months prior	No	Bilateral adrenalectomy	No	Approx. 2 months	Not stated
Hall (37)	1968	63	Metastatic	36 months prior	No	No	No	2 months	Coma, hypotension
Newmark (26)	1973	57	Metastatic	48 months prior	Yes	Mitotane, metyrapone	No	8 days	Sepsis
Lovern (24)	1975	66	Metastatic	48 months prior	No	Bilateral adrenalectomy	No	3 months	Liver failure secondary metastatic disease
Wenk (27)	1977	62	Locally advanced	8 months prior	No	No	No	23 days	Sepsis
Molland (38)	1978	76	Locally advanced	No	Not stated	Not stated	No	4 weeks	Coma? cause
Statham (39)	1981	63	Metastatic	36 months prior	No	Metyrapone	No	2 months	Renal failure
Vuitch (28)	1981	70	Metastatic	23 months prior	Not stated	Not stated	Not stated	2 months	Aspergillus
Carey (35)	1984	66	Metastatic	No	Not stated	Not stated	Doxorubicin, cyclophosphamide*	22 days	Bronchopneumonia
Ghali (29)	1984	76	Metastatic	No	No	Mitotane	No	6 weeks	Bronchopneumonia
Slater (30)	1985	69	Locally advanced	No	No	No	No	1 week	Sepsis
Fjellestad-Paulsen (36)	1988	63	Metastatic	No	No	Ketoconazole	No	6 days	Not stated
Haukaas (40)	1999	74	Metastatic	4 months prior	Yes	Metyrapone	No	80 days	Sepsis
Rickman (41)	2001	69	Metastatic	24 months prior	Yes	Ketoconazole	No	1 month	Not known
Hussein (31)	2002	53	Metastatic	9 months prior	Yes	Ketoconazole	No	2.5 months	Not stated
Nimalasena (42)	2008	64	Locally advanced	26 months prior	No	Ketoconazole, metyrapone	No	3.5 months	Not stated
Nimalasena (42)	2008	52	Metastatic	10 months prior	No	Ketoconazole, metyrapone, octreotide	Epirubicine, carboplatin, 5FU	1 month	Not stated
Alwani (25)	2009	62	Metastatic	No	No	Mifepristone, bilateral adrenalectomy	No	3 weeks	Sepsis
Alshaikh (32)	2010	70	Locally advanced	No	No	Ketoconazole, metyrapone	Cisplatin, etoposide	6 months	Sepsis
Rueda-Camino (17)	2016	63	Metastatic	No	No	Ketoconazole	Cisplatin, etoposide	12 months	Brain metastases
Shrosbree (33)	2016	71	Metastatic	Not stated	No	Metyrapone	Carboplatin, etoposide	Not stated	Not stated
Ramalingam (34)	2016	51	Metastatic	2 months prior	Yes	Ketoconazole	Cisplatin, etoposide	12 months	Not stated
Balestrieri (15)	2016	75	Locally advanced	No	Yes	Ketoconazole, metyrapone, octreotide	No	3 months	Intestinal perforation
Current case		71	Metastatic	No	Yes	Ketoconazole, metyrapone, octreotide	Carboplatin, etoposide	9 months	Metastatic disease

* Died shortly after first dose.

AdenoCa, prostate adenocarcinoma; CS, Cushing’s syndrome; dx, diagnosis; hx, history; MR, mineralocorticoid.

Of the reported cases of CS associated with SCPC most are due to ectopic ACTH production (24, 25, 26, 27, 28, 29, 30, 31, 32, 33, 34), but two cases of ectopic CRH production have also been reported (35, 36). As expected, based on the SCPC literature, in 14 of 25 cases, conventional prostate adenocarcinoma had been diagnosed a median of 23.5 months prior to the presentation with CS-associated SCPC (Table 2). While the current case also had concurrent prostate adenocarcinoma, the patient reported previous prostate surgery elsewhere and despite extensive efforts, the histology result was not available.

## Conclusions

SCPC is a rare but important cause of EAS. If not diagnosed and treated aggressively it is rapidly fatal. This case reminds us of the importance of detailed radiology review looking for evidence of ACTH-secreting lesions, consideration of a pelvic source and the need for aggressive treatment of hypercortisolism.

## Declaration of interest

The authors declare that there is no conflict of interest that could be perceived as prejudicing the impartiality of this review.

## Funding

This work did not receive any specific grant from any funding agency in the public, commercial, or not-for-profit sector.
